# Generality and Context-Specificity of Executive Function in College Students with Bullying Victimization Experiences

**DOI:** 10.3390/bs16091509

**Published:** 2026-08-27

**Authors:** Peng Li, Yu Gu, Mengmeng Zhao, Shuying Fu

**Affiliations:** 1 Faculty of Psychology, Tianjin Normal University, Tianjin 300387, China; lipeng@tjnu.edu.cn (P.L.);; 2 Hebei Key Laboratory of Mental Health and Brain Science, North China University of Science and Technology, Tangshan 063210, China; 3 Jiangsu Provincial University Key Lab of Child Cognitive Development and Mental Health, Yancheng Teachers University, Yancheng 224002, China

**Keywords:** bullying victimization, inhibitory control, working memory, context-specificity

## Abstract

Although previous research has confirmed an association between bullying victimization and executive function (EF) impairments, the long-term trajectory of these deficits remains controversial, specifically whether they persist as permanent scars into adulthood or are subject to a degree of neuroplastic recovery through brain maturation and subsequent learning experiences. Moreover, prior studies have largely focused on relatively stable, cross-task deficits, with limited examination of whether EF performance is dynamically modulated by victimization-related contextual cues. Accordingly, the present study examined the differences in EF performance among individuals with a history of bullying victimization under both general processing conditions and context-induced conditions through two experiments. Experiment 1a and Experiment 2a assessed general inhibitory control (IC) and working memory (WM) using the classic Stroop and N-back paradigms, respectively. Experiment 1b and Experiment 2b introduced victimization-related contextual cues into these paradigms to examine context-dependent alterations in cognitive control. The results showed that, under general conditions, the victimization group exhibited significantly longer reaction times (RTs) than the control group in both the Stroop and N-back tasks, indicating less efficient general cognitive processing. Further analyses revealed distinct patterns under context-specific conditions: in the adapted Stroop paradigm, the victimization group showed relatively faster RTs in victimization-related contexts, suggesting context-dependent differences in interference control; in contrast, in the WM task, RTs were prolonged in victimization-related contexts, suggesting greater contextual interference during WM processing. In conclusion, bullying victimization is associated not only with general differences in EF performance among college students but also with context-dependent alterations in cognitive processing. These findings highlight the context-dependent effects of bullying-related cues on EF and provide behavioral evidence that bullying victimization is associated with alterations in cognitive control processes, with potential implications for developing targeted intervention strategies.

## 1. Introduction

Bullying victimization refers to aggressive behaviors in which an individual is intentionally harmed by others, characterized by repetitiveness, intent, and an imbalance of power (Olweus, 1994). As a severe form of interpersonal trauma, early experiences of victimization not only inflict immediate harm but also produce significant long-term and latent effects, acting like a psychological scar that is difficult to heal (Lewinsohn et al., 1981; Jungup, 2020; Young et al., 2006). Research indicates that traumatic experiences can alter an individual’s psychological and cognitive developmental trajectories. Even years after the bullying has ceased, its negative impacts remain latent and persistent, significantly increasing the risk of both internalizing and externalizing behavioral problems in adulthood (Hwang et al., 2017). These include depressive symptoms (Zhao et al., 2024), alexithymia (Wen et al., 2026), low self-esteem (Kangkana & Manjula, 2017), aggression (Young et al., 2006), and even suicide (Shi et al., 2020).

Executive function (EF) refers to a top-down cognitive control process that involves the dynamic and flexible integration of multiple cognitive subsystems to coordinate complex activities in the pursuit of goal-directed behavior. Although EF is commonly conceptualized as comprising three core components, namely inhibitory control (IC), working memory (WM), and cognitive flexibility (CF) (Miyake et al., 2000; Doebel, 2020), these components jointly support adaptive cognitive regulation and enable individuals to maintain goal-directed behavior in the face of internal and external demands. Among these components, IC and WM are considered foundational processes underlying EF. Importantly, IC is not a unitary ability but encompasses both response inhibition and interference control (Diamond, 2013). Response inhibition refers to the ability to suppress prepotent or automatic responses, whereas interference control involves resisting interference from task-irrelevant information while maintaining goal-relevant processing. Thus, IC encompasses multiple forms of cognitive control that support goal-directed behavior under conditions of competing responses or distracting information. In contrast, CF is generally regarded as a higher-order control function involving the ability to flexibly shift between different task sets and adapt behavior according to changing environmental demands (Davidson et al., 2006; Diamond, 2013). Given that the present study focuses on two foundational components of EF that may be particularly sensitive to the cognitive consequences of victimization, we specifically examine IC and WM.

Extensive research has confirmed a close association between bullying victimization and impairments in EF (Lu et al., 2025; Potard et al., 2021). Victimized children and adolescents perform significantly worse than their non-victimized peers on the Stroop task, Flanker task, and N-back task, as manifested by reduced IC efficiency and decreased WM capacity. Longitudinal studies have further indicated that peer victimization in childhood predicts the developmental level of EF in adolescence and even early adulthood (Holmes et al., 2016; Patricia et al., 2025). However, findings regarding the impact of bullying victimization on EF remain inconsistent. Some studies have reported stable and significant EF deficits, whereas others have found no significant differences or have observed impairments only under specific task conditions (Augusti & Melinder, 2013; Liu & Hao, 2015). Such inconsistencies suggest that the effects of victimization on EF may not be fixed but may instead be moderated by factors such as task type and stimulus context.

Two primary theoretical frameworks have been proposed to account for these effects, namely neurodevelopmental theory and self-regulation theory. The neurodevelopmental perspective posits that chronic social stress during childhood disrupts the normal functioning of the hypothalamic–pituitary–adrenal (HPA) axis, which in turn adversely affects the development of prefrontal cortex and other brain regions subserving EF, ultimately leading to enduring deficits in cognitive control (Andersen, 2003; Bremner et al., 2003; Peverill et al., 2023). Concurrently, self-regulation resource theory suggests that prolonged social exclusion and bullying continuously deplete individuals’ limited cognitive resources, undermining their capacity for effective emotional regulation and cognitive control (Baumeister et al., 2005, 1994). Nevertheless, these mechanisms primarily explain how the damage occurs, but leave unanswered the question of whether the damage is permanent. Given that prefrontal development extends into early adulthood, existing theories diverge fundamentally on whether early-life impairments naturally subside with brain maturation or become consolidated into long-lasting functional deficits. On one hand, neuroplasticity theory maintains that the brain retains the capacity for ongoing reorganization and functional recovery and that learning and training in adulthood may ameliorate cognitive deficits arising from early adversity (Hebb, 2005). On the other hand, the scar theory contends that early trauma leaves persistent cognitive imprints; even when overt behavior appears to have normalized, latent effects may endure and re-emerge under specific circumstances (Lewinsohn et al., 1981). Accordingly, examining the long-term consequences of bullying victimization during early adulthood when EF has reached relative maturity and stability not only helps ascertain whether such deficits persist, but also facilitates differentiation between developmental delay and sustained post-traumatic sequelae.

Beyond the aforementioned theoretical debate concerning temporal persistence, the manifestation of victimization effects on EF also exhibits complexity. A growing body of research has highlighted that EF is characterized not only by general stability across tasks, but also by marked contextual dependence (Miller et al., 2023). General EF reflects an individual’s relatively stable cognitive control ability across diverse tasks, whereas context-specific EF emphasizes that cognitive control processes dynamically shift in response to environmental stimuli, particularly emotional or threat-related cues (Reineberg et al., 2021). Studies have found that victimized children demonstrate poorer WM and IC in bullying-relevant contexts (Potard et al., 2021), suggesting that victimization may not only impair general EF, but may also alter cognitive processing patterns in trauma-related situations. However, existing evidence relies heavily on questionnaire-based measures, lacking verification through objective behavioral paradigms. Moreover, most studies have focused on children and adolescents, leaving a notable gap in systematic research on whether similar context-specific processing characteristics persist into adulthood.

In summary, the present study employs behavioral experimental paradigms to systematically examine general and context-specific EF performance in college students with a history of bullying victimization. Experiment 1 employs the classic Stroop paradigm and an adapted Stroop-like paradigm to examine general inhibitory control and context-dependent interference control, respectively. Experiment 2 employs the classic N-back paradigm and an adapted version incorporating victimization-related contextual cues to examine general WM and context-dependent WM performance, respectively. This study seeks to examine whether a history of bullying victimization is associated with general EF differences and whether victimization-related contextual cues modulate EF performance in context-dependent situations.

## 2. Materials and Methods

### 2.1. Participants

Participants were recruited using cluster sampling, with classes serving as the sampling units. Undergraduate students at a university were administered the Retrospective Bullying Scale (Rivers, 2001), yielding a total of 3589 valid questionnaires. Based on the total scores, participants were ranked from highest to lowest, and individuals were then randomly selected from the extreme ends of the distribution to form the victimization group and the control group.

Experiment 1a and Experiment 1b were completed by the same group of participants, with Experiment 1b administered after Experiment 1a. Similarly, Experiment 2a and Experiment 2b were completed by the same group of participants. However, the samples recruited for Experiment 1 and Experiment 2 were independent, and no participants participated in both experiments. For each task, data quality was evaluated separately according to predefined exclusion criteria. Therefore, the final analyzable sample sizes differed slightly across paired tasks. Specifically, participants were retained for a given task if their data met the task-specific inclusion criteria, whereas their data were excluded from that task analysis if they exceeded the predefined exclusion thresholds. Thus, differences in valid sample sizes across tasks reflect task-level data exclusion rather than differences in participant recruitment. Descriptive statistics for participant characteristics and RBQ scores in Experiments 1 and 2 are presented in Table 1.

A total of 65 participants were recruited for Experiment 1. Participants who failed to correctly understand the task instructions or exhibited an overall error rate greater than 30% were excluded from the analyses. Following recommendations for reaction time (RT) data processing (Whelan, 2008) and previous studies using the Stroop and N-back paradigms (Goldfarb & Henik, 2013; Vytal et al., 2012), trials with RTs shorter than 200 ms were classified as anticipatory responses and excluded, as such responses are unlikely to reflect complete stimulus processing. To minimize the influence of excessively delayed responses resulting from attentional lapses or temporary disengagement from the task, trials with RTs longer than 3000 ms were also excluded. In Experiment 1a, three participants suspended the experiment due to practical circumstances, and two were excluded due to poor performance. The final valid sample consisted of 31 participants in the victimization group and 29 in the control group. In Experiment 1b, the final valid sample included 34 participants in the victimization group and 31 in the control group.

Similarly, a total of 61 participants were recruited for Experiment 2. Following the same exclusion criteria as in Experiment 1, the final sample for Experiment 2a consisted of 31 participants in the victimization group and 30 participants in the control group. In Experiment 2b, five participants discontinued the experiment due to practical reasons; thus, the final sample comprised 28 participants in the victimization group and 28 participants in the control group.

All participants were college students, right-handed, had normal intelligence, and reported normal or corrected-to-normal vision. In addition, to minimize the potential influence of clinically significant psychopathology on EF and to more accurately isolate the association between bullying victimization and EF, individuals who self-reported a diagnosis of a major psychiatric disorder were excluded. All participants volunteered to participate, provided written informed consent prior to the experiment, and received monetary compensation upon completion of the study.

### 2.2. Instruments

#### 2.2.1. Retrospective Bullying Questionnaire (RBQ)

The Retrospective Bullying Questionnaire (RBQ) was originally developed by a collaborative research team from Germany, Spain, and the United Kingdom based on the Secondary-school Bullying Questionnaire (SBQ; Rivers, 2001) to retrospectively assess experiences of bullying victimization. In the present study, only the bullying victimization items were administered. A Chinese version of the RBQ developed and culturally adapted by our research team was used. The adaptation followed standard translation and cultural adaptation procedures and was psychometrically evaluated in a large sample of Chinese university students, demonstrating satisfactory factorial validity, internal consistency, test–retest reliability, criterion-related validity, and measurement invariance. The adapted questionnaire consists of 21 items across four dimensions. Each item was rated on a 5-point Likert scale, and the score for each dimension was calculated by summing the corresponding item scores. In the current study, the scale demonstrated excellent internal consistency (Cronbach’s α = 0.931). Confirmatory factor analysis (CFA) also supported the four-factor structure, yielding an acceptable model fit (*χ*^2^*/df* = 4.786, CFI = 0.975, TLI = 0.968, SRMR = 0.040, RMSEA = 0.052). See Table S1 in Supplementary Materials.

#### 2.2.2. Experimental Materials

Color-word stimuli: In the classic Stroop task, the stimuli consisted of the Chinese characters “yellow” and “green”, which were randomly presented in either yellow or green ink, with the number of trials balanced across conditions. In the adapted Stroop task, yellow and blue color frames were selected and balanced across the three contexts, ensuring that the number of combinations between the color frames and the contexts was identical.

**Figure 1:**
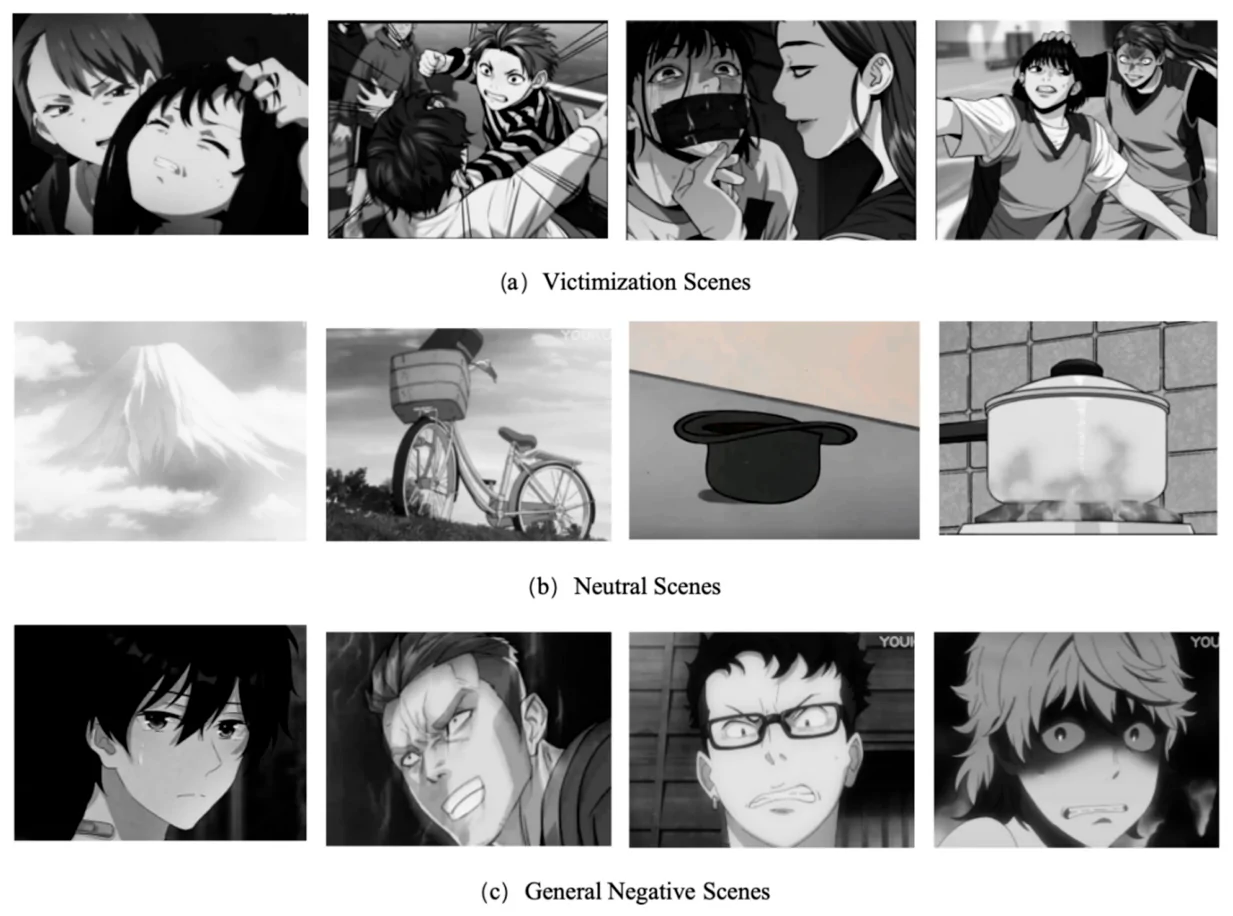
Examples of experimental materials.

Picture stimuli: To determine whether the impact of bullying victimization on EF is specific to bullying-related contexts, this study selected three types of cartoon-style images: (a) victimization scenes, (b) general negative scenes, and (c) neutral scenes. Cartoon-style images were adopted instead of photographs of real individuals to minimize potential psychological distress while preserving the essential social meaning of the bullying scenarios, following previous studies using similar visual materials (Li et al., 2023; Ratajska et al., 2020). Initially, 60 images were selected for each of the three categories, as shown in Figure 1. Participants were asked to identify the category of each image and rate them on a 1-to-5 scale for valence and arousal. Based on these ratings, a final set of 30 images (10 per category) was selected. The descriptive statistics for the ratings are presented in Table 2.

#### 2.2.3. Experimental Design

The present study consisted of two experiments designed to examine the generality and context specificity of EF impairments associated with bullying victimization. Experiment 1 investigated IC, whereas Experiment 2 examined WM. Each experiment included a general cognitive task and an adapted task incorporating bullying-related contextual stimuli to determine whether EF differences were domain-general or context-dependent.

Experiment 1 included two tasks assessing IC. Experiment 1a employed the classic Stroop task to measure general IC. A 2 (Group: victimization vs. control) × 2 (Congruency: congruent vs. incongruent) mixed design was adopted, with Group as the between-subjects factor and Congruency as the within-subjects factor. Experiment 1b further examined context-specific interference control by incorporating emotional contextual stimuli into the Stroop paradigm. A 2 (Group: victimization vs. control) × 3 (Stimulus Context: victimization, general negative, and neutral) mixed design was employed, with Group as the between-subjects factor and Stimulus Context as the within-subjects factor.

Experiment 2 investigated WM using the n-back paradigm. Experiment 2a employed a traditional digit-based n-back task to assess general WM ability. A 2 (Group: victimization vs. control) × 2 (Task Difficulty: low vs. high) mixed design was used, with Group as the between-subjects factor and Task Difficulty as the within-subjects factor. Task difficulty was manipulated by varying WM load: the 1-back condition represented the low-load condition, whereas the 2-back condition represented the high-load condition. Experiment 2b extended the n-back paradigm by replacing neutral digit stimuli with emotional contextual stimuli to examine context-specific WM processing. A 2 (Group: victimization vs. control) × 2 (Task Difficulty: low vs. high) × 3 (Stimulus Context: victimization, general negative, and neutral) mixed design was adopted.

Across all experiments, participants’ RTs and accuracy were recorded as behavioral indicators. RTs from correct responses were considered the primary measure of processing efficiency.

#### 2.2.4. Experimental Procedure

All experimental procedures were programmed using E-Prime 2.0 (Psychology Software Tools, Inc., Sharpsburg, PA, USA) and conducted in a quiet laboratory environment. Before each formal task, participants completed practice trials to familiarize themselves with the experimental procedures and response requirements. The assignment of response keys was counterbalanced across participants to control for potential effects of response mapping.

**Figure 2:**
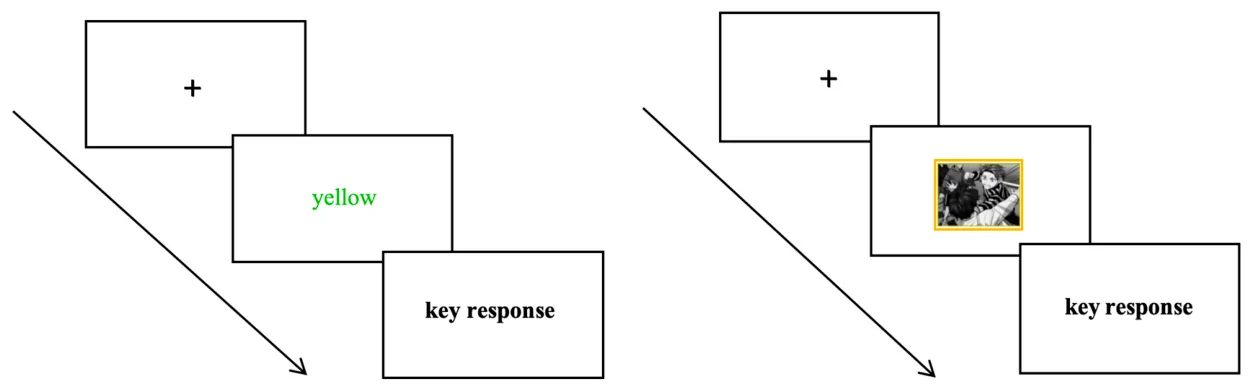
Experimental procedure flowchart for Experiment 1.

Experiment 1: Experiment 1a used the classic word-color Stroop task. After reading the instructions, participants were required to identify the ink color of the presented Chinese characters as quickly and accurately as possible. Each trial began with a fixation cross (“+”) presented at the center of the screen for 500 ms, followed by the target stimulus. Participants responded by pressing the corresponding key according to the ink color of the character. The next trial started immediately after the response was recorded. After completing Experiment 1a, participants were given a 5-min break before proceeding to Experiment 1b. The procedure of Experiment 1b was similar to that of Experiment 1a, except that contextual emotional pictures were presented as background cues. Participants were instructed to identify the color of the frame surrounding each picture while ignoring the semantic content of the picture. The experimental procedure of Experiment 1 is illustrated in Figure 2.

**Figure 3:**
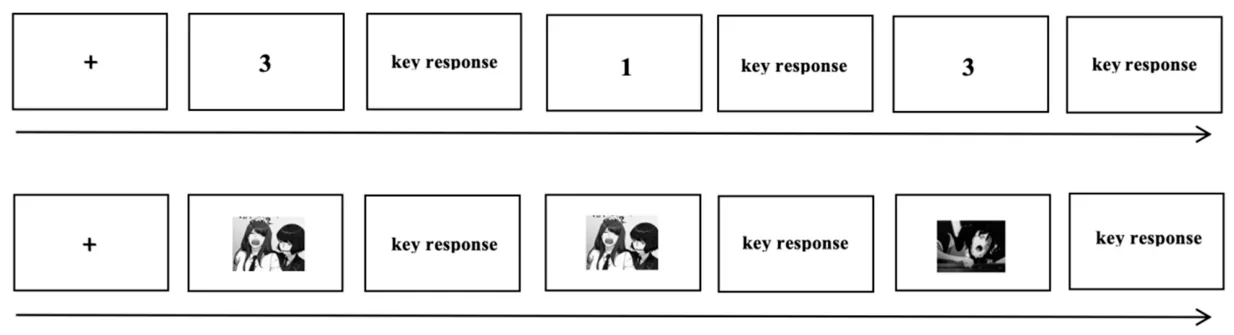
Experimental procedure flowchart for Experiment 2.

Experiment 2: Experiment 2a employed the classic n-back task. In the 1-back condition (*n* = 1), participants were required to judge whether the current digit was the same as the immediately preceding one, pressing the “V” key for a match and the “N” key for a non-match. In the 2-back condition (*n* = 2), participants judged whether the current digit matched the one presented two positions back, pressing “V” for a match and “N” for a non-match. Each digit was presented for 500 ms, followed by a 3000 ms response window. The assignment of the “V” and “N” keys was counterbalanced across participants. After this task, participants took a 5-min break before proceeding to Experiment 2b. This task replaced the digits with the emotional context pictures, requiring participants to respond based on whether consecutive pictures were identical. The practice block consisted of 32 trials, and the formal experiment consisted of 64 trials. Upon completion, participants were thanked and given the corresponding compensation. The experimental procedures are illustrated in Figure 3.

#### 2.2.5. Data Analysis

Statistical analyses were conducted using IBM SPSS Statistics 24.0. Behavioral data were preprocessed according to the exclusion criteria described above. RT for correct responses served as the primary dependent variable in all experiments. Response accuracy was also analyzed to examine whether any RT effects were accompanied by speed–accuracy trade-offs.

For Experiment 1a, a 2 (Group: victimization vs. control) × 2 (Congruency: congruent vs. incongruent) mixed-design repeated-measures ANOVA was conducted, with Group as the between-subjects factor and Congruency as the within-subjects factor. For Experiment 1b, a 2 (Group: victimization vs. control) × 3 (Stimulus Context: victimization, general negative, and neutral) mixed-design repeated-measures ANOVA was conducted, with Group as the between-subjects factor and Stimulus Context as the within-subjects factor. For Experiment 2a, a 2 (Group: victimization vs. control) × 2 (Task Difficulty: 1-back vs. 2-back) mixed-design repeated-measures ANOVA was conducted, with Group as the between-subjects factor and Task Difficulty as the within-subjects factor. For Experiment 2b, a 2 (Group: victimization vs. control) × 2 (Task Difficulty: 1-back vs. 2-back) × 3 (Stimulus Context: victimization, general negative, and neutral) mixed-design repeated-measures ANOVA was conducted.

When significant interaction effects were detected, Bonferroni-corrected simple effects analyses were performed for further comparisons. Mauchly’s test was conducted to examine the assumption of sphericity, and Greenhouse–Geisser corrections were applied when the assumption was violated. Effect sizes were reported as partial eta squared (*ηp*^2^), with *ηp*^2^ values of 0.01, 0.06, and 0.14 interpreted as small, medium, and large effects, respectively (Cohen, 2013). Statistical significance was determined at *p* < 0.05.

## 3. Results

### 3.1. General and Context-Specific Inhibitory Control

Results of the General IC (Experiment 1a): A repeated-measures ANOVA was conducted on the RTs. The results revealed a significant main effect of Group (*F* = 7.03, *p* = 0.01, *ηp*^2^ = 0.11), with the victimization group showing significantly longer RTs than the control group. There was also a significant main effect of Congruency (*F* = 23.69, *p* < 0.001, *ηp*^2^ = 0.29), where RTs in the congruent condition were significantly shorter than those in the incongruent condition. However, the interaction between Group and Congruency was not significant (*F* = 3.48, *p* = 0.07, *ηp*^2^ = 0.06). The results are presented in Table 3.

Similarly, accuracy was analyzed using a repeated-measures ANOVA. The results revealed a significant main effect of Congruency, *F* = 8.31, *p* = 0.01, *ηp*^2^ = 0.13, with higher accuracy in the congruent condition (0.96 ± 0.09) than in the incongruent condition (0.94 ± 0.12). The main effect of Group and the Group × Congruency interaction were not significant (both *ps* > 0.05).

**Figure 4:**
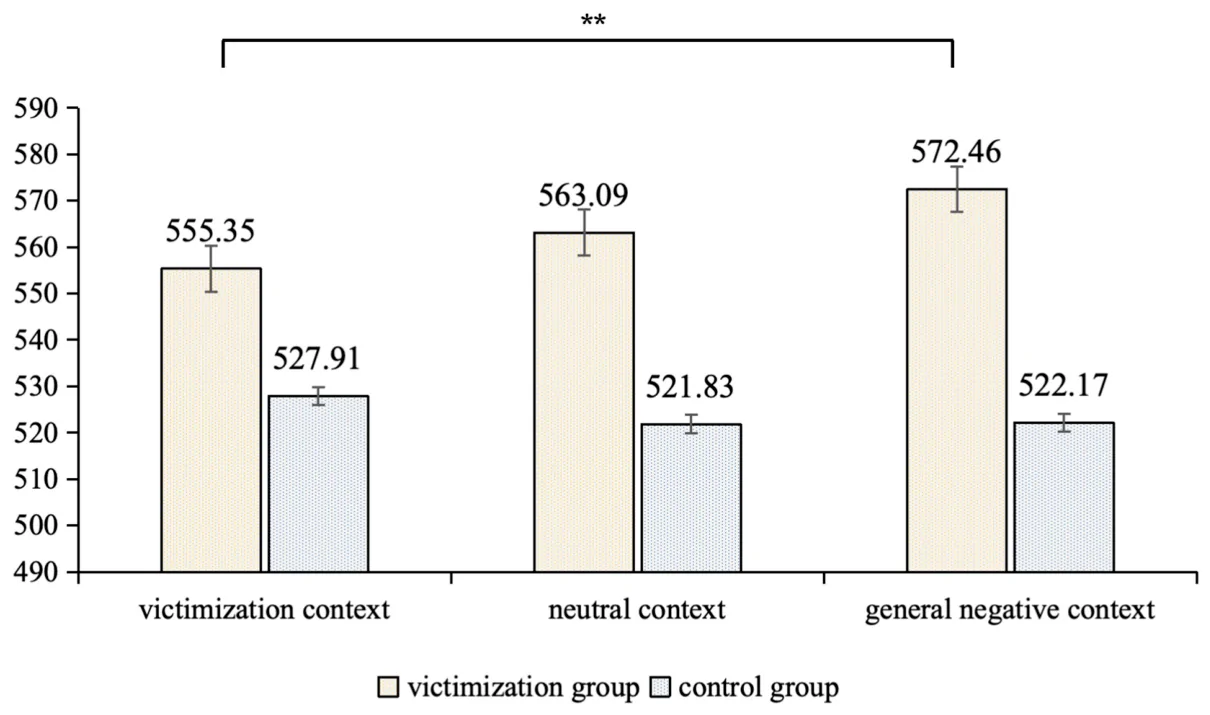
Mean reaction times of groups across different stimulus contexts. Note: ** *p* < 0.01.

Results of Context-Specific IC (Experiment 1b): A repeated-measures ANOVA was conducted on RTs. The results showed that neither the main effect of Group nor the main effect of Stimulus Context was significant (all *ps* > 0.05). However, the interaction between Group and Stimulus Context was significant, *F* = 3.08, *p* = 0.05, *ηp*^2^ = 0.05. Therefore, simple effects analyses were conducted to further examine this interaction. The results showed that RTs differed significantly across stimulus contexts within the victimization group (*p* = 0.01). Specifically, the victimization group exhibited significantly faster RTs in the victimization-related context than in the general negative context. These findings indicate that individuals with bullying victimization experiences responded more quickly when processing victimization-related cues compared with general negative cues. The results are presented in Table 4 and Figure 4.

And accuracy analyses revealed no significant effects of Group, Stimulus Context, or their interaction (all *p*s > 0.05).

### 3.2. General and Context-Specific Working Memory

Results of the General WM (Experiment 2a): A repeated-measures ANOVA was conducted on the RTs. The results revealed a significant main effect of Group (*F* = 4.42, *p* = 0.04, *ηp*^2^ = 0.07), with the victimization group showing significantly longer RTs than the control group. There was also a significant main effect of Task Difficulty (*F* = 31.75, *p* < 0.001, *ηp*^2^ = 0.35), where RTs in the low-task-difficulty condition were significantly shorter than those in the high-task-difficulty condition. However, the interaction between Group and Task Difficulty was not significant (*p* = 0.99 > 0.05).

Similarly, accuracy was analyzed using a repeated-measures ANOVA. The results revealed a significant main effect of Task Difficulty, *F* = 33.99, *p* < 0.001, *ηp*^2^ = 0.37, with higher accuracy in the low-task-difficulty condition than in the high-task-difficulty condition. A significant main effect of Group was also observed, *F* = 4.31, *p* = 0.04, *ηp*^2^ = 0.07, indicating that the control group exhibited significantly higher accuracy than the victimization group. The Group × Task Difficulty interaction was not significant (*p* > 0.05). The results are presented in Table 5.

Results of Context-Specific WM (Experiment 2b): A repeated-measures ANOVA was conducted on RTs. The results showed that the three-way interaction among Group, Task Difficulty, and Stimulus Context was not significant (*p* > 0.05). Neither the Group × Task Difficulty interaction nor the Task Difficulty × Stimulus Context interaction was significant (both *ps* > 0.05). However, a significant Group × Stimulus Context interaction was observed, *F* = 3.29, *p* = 0.04, *ηp*^2^ = 0.06. In addition, there was a significant main effect of Task Difficulty, *F* = 29.03, *p* < 0.001, *ηp*^2^ = 0.35, with RTs being significantly shorter under the low-load condition than under the high-load condition. Neither the main effect of Group nor that of Stimulus Context reached statistical significance (both *ps* > 0.05).

**Figure 5:**
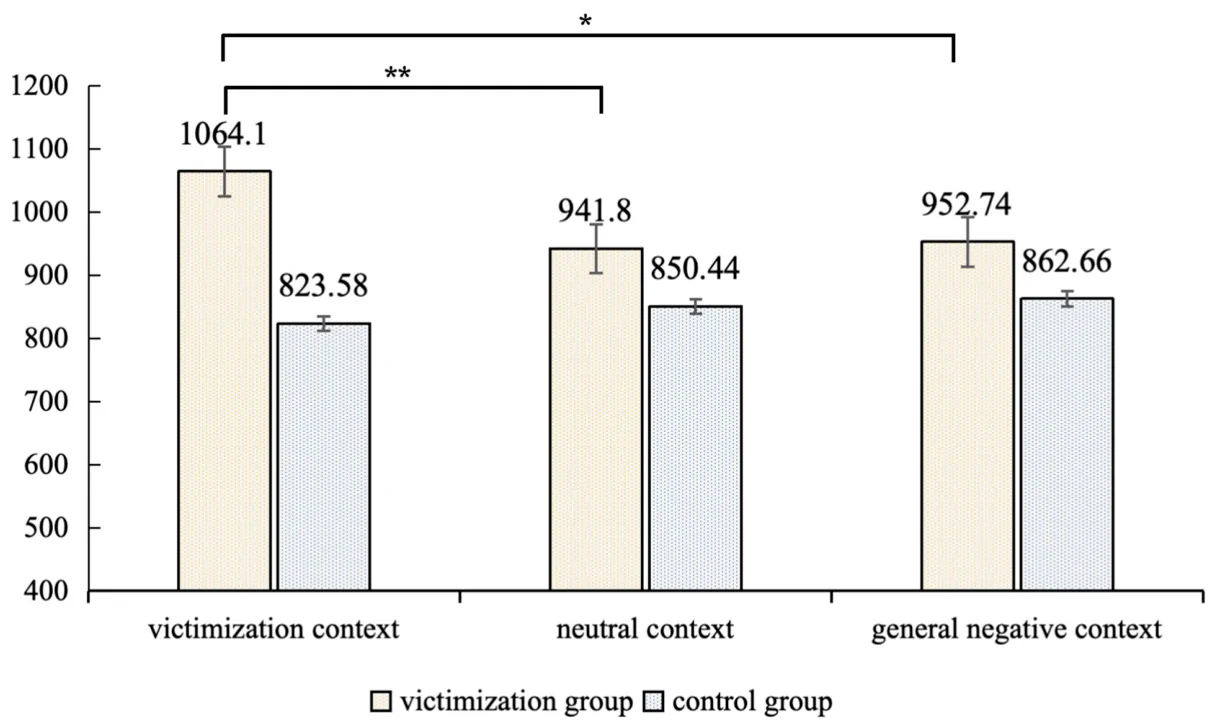
Mean reaction times across groups and stimulus contexts. Note: ** *p* < 0.01, * *p* < 0.05.

Because the Group × Stimulus Context interaction was significant, simple effects analyses were conducted. The results revealed a significant effect of Stimulus Context within the victimization group (*p* = 0.01). Specifically, participants in the victimization group exhibited significantly longer RTs in the victimization-related context than in both the general negative and neutral contexts. No significant differences in RTs across stimulus contexts were observed in the control group (see Table 6 and Figure 5).

Similarly, accuracy analyses revealed no significant effects of Group, Stimulus Context, Task Difficulty or their interaction (all *ps* > 0.05).

## 4. Discussion

The present study was designed to address two unresolved questions regarding EF following bullying victimization. First, whether EF differences associated with bullying victimization persist into adulthood, as proposed by the scar hypothesis, or are attenuated through subsequent neuroplastic recovery. Second, whether these cognitive differences represent generalized EF characteristics or vary according to bullying-related contextual cues. By integrating classic and context-adapted Stroop and N-back paradigms, the present findings provide behavioral evidence that EF differences associated with bullying victimization remain observable in college students under general conditions, lending support to the scar hypothesis. At the same time, the findings further demonstrate that these EF differences are not entirely generalized but are modulated by bullying-related contextual information. Together, these findings suggest that the long-term cognitive correlates of bullying victimization involve both generalized EF differences and context-dependent variations in cognitive control performance.

The results of Experiments 1a and 2a showed that participants with bullying victimization experiences exhibited significantly longer reaction times than the control group in both the classic Stroop and N-back tasks, indicating poorer IC and WM performance under general conditions. These findings are generally consistent with previous studies reporting an association between bullying victimization and EF difficulties (Mara & François, 2018; Juvonen & Graham, 2014) and suggest that EF differences associated with bullying victimization may remain observable into early adulthood. This pattern is more consistent with the scar hypothesis, which proposes that adverse interpersonal experiences may have enduring cognitive consequences, than with the view that such differences are fully attenuated through subsequent neuroplastic recovery. The reaction time findings were generally supported by the accuracy results. In the WM task, participants with bullying victimization experiences also exhibited lower accuracy than the control group, whereas no group differences in accuracy were observed in the IC task. This pattern suggests that the observed reaction time differences are unlikely to reflect a simple speed–accuracy trade-off. Interestingly, group differences in IC were reflected primarily in processing efficiency (i.e., slower responses), whereas WM differences were evident in both response speed and accuracy, suggesting that WM may be more vulnerable to the long-term cognitive correlates associated with bullying victimization. One possible explanation is that repeated exposure to stressful interpersonal experiences may continuously consume cognitive resources required for executive control, thereby reducing processing efficiency during cognitively demanding tasks (Xu et al., 2025; Anyerson et al., 2024). In addition, childhood and adolescence represent critical periods for EF development. Previous neurodevelopmental research has suggested that chronic stress associated with adverse experiences may influence the maturation of brain regions supporting executive control, particularly the prefrontal cortex (Margit et al., 2011; Kang et al., 2022). Although the present cross-sectional findings cannot establish causal relationships, they are consistent with the possibility that bullying victimization is associated with persistent differences in EF performance that remain evident in adulthood.

The results of Experiment 1b showed a significant interaction between Group and Stimulus Context. Further simple effects analyses revealed that participants in the victimization group exhibited significantly faster RTs in the victimization-related context than in the general negative context, whereas the control group showed no significant RT differences across stimulus contexts. Notably, no corresponding differences in response accuracy were observed, suggesting that the RT pattern was unlikely to reflect a simple speed–accuracy trade-off. These findings suggest that bullying victimization experiences may be associated with context-dependent differences in cognitive control performance. Rather than representing a conventional Stroop effect based on response conflict, the adapted paradigm was designed to examine context-dependent interference control by presenting victimization-related images as task-irrelevant but socially salient distractors while participants maintained attention to the color-frame judgment task. Thus, the observed RT differences may reflect differences in the extent to which victimization-related contextual cues influence goal-directed processing, rather than differences in classical response inhibition. The relatively faster responses observed in the victimization group in the victimization-related context may reflect a distinct pattern of information processing when individuals encounter cues associated with their previous bullying experiences. Repeated exposure to negative social interactions may increase the salience of bullying-related information, thereby influencing the allocation and regulation of attention between task-relevant and task-irrelevant information in socially threatening contexts. The vigilance-avoidance model (Mogg et al., 2004) provides one possible theoretical framework for understanding why bullying-related cues may differentially influence cognitive processing in individuals with bullying victimization experiences. According to this model, threat-related stimuli may initially capture attention before subsequent attentional regulation occurs. However, because the present study assessed only overall behavioral performance, it cannot determine whether the observed RT differences reflect enhanced threat sensitivity, attentional regulation, attentional capture, or other underlying cognitive processes. Moreover, the observed interaction was associated with a relatively modest effect size (*ηp*^2^ = 0.05). Therefore, the present findings should be interpreted as preliminary evidence that bullying-related contextual cues may modulate context-dependent interference control rather than as direct evidence for impaired IC or a specific attentional mechanism.

In contrast to the IC findings, Experiment 2b showed that participants in the victimization group exhibited significantly longer RTs in the victimization-related context than in the neutral and general negative contexts, whereas no corresponding differences were observed in response accuracy. This pattern suggests that bullying-related contextual cues may impose greater cognitive demands during WM processing without producing measurable changes in task accuracy. In the adapted N-back task, contextual pictures served as task-relevant stimuli that participants were required to encode, maintain, and continuously update in WM. Unlike the adapted Stroop task, in which contextual images functioned as task-irrelevant distractors, participants could not ignore bullying-related information because successful task performance depended on actively processing these stimuli. Consequently, bullying-related contextual cues may have generated greater cognitive interference during WM processing. This interpretation is broadly consistent with processing efficiency theory (Eysenck & Calvo, 1992), which proposes that emotionally salient information competes for limited executive resources, thereby reducing processing efficiency during cognitively demanding tasks. In the present study, bullying-related contextual cues may have required additional cognitive resources during WM processing, contributing to slower behavioral responses. Importantly, the significant Group × Stimulus Context interaction was associated with a relatively modest effect size (*ηp*^2^ = 0.06). Therefore, although the findings suggest that bullying-related contextual cues may differentially influence WM performance, the underlying cognitive processes cannot be determined from behavioral measures alone and require further investigation using methods capable of directly assessing attentional and neural processing. However, given the limited sample size and the exploratory nature of this analysis, this finding should be interpreted cautiously and requires replication.

Taken together, the present findings extend the current understanding of EF associated with bullying victimization in several important ways. Rather than supporting a unitary deficit account, the present study suggests that EF associated with bullying victimization comprises both generalized differences that are observable across conventional cognitive tasks and context-dependent alterations that emerge when individuals process bullying-related social cues. This integrated perspective may help explain why previous studies have reported inconsistent findings regarding EF following bullying victimization, as task context may substantially influence the manifestation of cognitive differences across EF components. These findings also have important practical implications. The distinction between generalized and context-dependent EF characteristics suggests that intervention strategies may need to target different aspects of cognitive functioning. General EF differences may benefit from cognitive training designed to improve IC and WM, whereas context-dependent cognitive alterations highlight the importance of trauma-informed interventions that strengthen emotion regulation and adaptive processing of bullying-related social information. Such approaches may help reduce the influence of bullying-related contextual cues on cognitive performance and promote more adaptive functioning in everyday social environments.

Meanwhile, the present study has several limitations that should be considered when interpreting the findings. First, although the present study examined two core components of EF, CF, another important component of EF, was not assessed. Future studies should examine all three EF components to provide a more comprehensive understanding of the cognitive consequences of bullying victimization. Second, the relatively small sample size may have limited the statistical power to detect complex interaction effects, particularly higher-order interactions. Therefore, the interaction effects involving multiple factors should be interpreted cautiously and require replication with larger samples. Third, participants’ familiarity with anime-style materials was not assessed. Although cartoon-style stimuli were used to minimize potential psychological distress associated with realistic bullying scenes, familiarity differences may have influenced participants’ responses. Future studies should further validate the findings using alternative types of stimuli. Finally, the present study relied primarily on behavioral measures and did not assess several psychological factors that may influence both bullying victimization and EF. Future studies combining behavioral and neurophysiological measures while considering relevant psychological factors may further clarify the mechanisms underlying the relationship between bullying victimization and EF.

## 5. Conclusions

The present study demonstrates that EF associated with bullying victimization is characterized by both generalized differences under conventional cognitive tasks and context-dependent alterations in response to bullying-related social cues. These findings suggest that EF following bullying victimization should not be viewed as a unitary cognitive deficit but rather as a context-sensitive pattern of cognitive functioning. By integrating general and context-specific experimental paradigms across IC and WM, the present study provides a more comprehensive understanding of the long-term cognitive correlates associated with bullying victimization and offers a behavioral framework for future research examining the mechanisms underlying these cognitive differences.

## Figures and Tables

**Table 1:** Descriptive Statistics of Participant Characteristics and Scores in Experiments 1 and 2.

	Age	Gender		Score Range	RBQ Score	*t*
		Male	Female			
Experiment 1						
Victimization group	18.81 ± 0.74	19	13	50–90	61.34 ± 9.84	16.01 ***
Control group	19.67 ± 1.14	11	22	21–43	28.79 ± 6.04	
Experiment 2						
Victimization group	18.77 ± 0.82	18	12	22–40	61.70 ± 11.13	14.49 ***
Control group	19.03 ± 1.25	10	21	53–95	29.03 ± 5.71	

Note: *** *p* < 0.001.

**Table 2:** Descriptive statistics of emotional picture dimension ratings (*M* ± *SD*).

Picture Category	Victimization Scenes	General Negative Scenes	Neutral Scenes	*F*
Valence	1.351 ± 0.293	1.933 ± 0.127	3.009 ± 0.178	176.894 ***
Arousal	4.417 ± 0.402	3.600 ± 0.276	2.739 ± 0.226	87.900 ***

Note: *** *p* < 0.001.

**Table 3:** Comparison of RTs and accuracy by group and congruency condition (*M* ± *SD*).

	Group	RTs (ms)	Accuracy
congruent	victimization group	526.17 ± 86.83	0.94 ± 0.12
	control group	477.80 ± 83.51	0.96 ± 0.09
incongruent	victimization group	563.15 ± 93.15	0.93 ± 0.13
	control group	494.30 ± 88.74	0.95 ± 0.09

**Table 4:** Comparison of RTs by group and stimulus context (*M* ± *SD*).

Group	Stimulus Context		
	Victimization Context	Neutral Context	General Negative Context
victimization group	555.35 ± 78.20	563.09 ± 87.25	572.46 ± 94.83
control group	527.91 ± 131.09	521.83 ± 119.16	522.17 ± 128.00

**Table 5:** Comparison of RTs and accuracy by group and task difficulty (*M* ± *SD*).

Task Difficulty	Group	RTs (ms)	Accuracy
low difficulty	victimization group	810.81 ± 399.19	0.91 ± 0.11
	control group	648.66 ± 294.13	0.96 ± 0.05
high difficulty	victimization group	1247.16 ± 505.42	0.85 ± 0.11
	control group	1081.85 ± 479.82	0.88 ± 0.79

**Table 6:** RTs by group across different difficulty levels and contexts (*M* ± *SD*, ms).

Task Difficulty	Group	Victimization Context	Neutral Context	General Negative Context
low difficulty	victimization group	902.79 ± 290.41	878.94 ± 336.77	790.70 ± 366.05
	control group	636.77 ± 302.38	639.77 ± 264.70	697.99 ± 381.58
high difficulty	victimization group	1225.42 ± 475.80	1004.65 ± 372.34	1114.79 ± 561.37
	control group	1010.40 ± 391.50	1061.10 ± 525.56	1027.33 ± 467.29

## Data Availability

The data that support the findings of this study are available from the corresponding authors upon reasonable request due to privacy.

## Supplementary Materials

The following supporting information can be downloaded at: https://www.mdpi.com/article/10.3390/bs16091509/s1, Supplementary Material S1. Retrospective Bullying Questionnaire (RBQ); Table S1. RBQ Items; Table S2. Reliability Analysis of RBQ Subscales (*N* = 3859).
