# Supplementary material for: Generality and Context-Specificity of Executive Function in College Students with Bullying Victimization Experiences
Source: Behav Sci (Basel). 2026 Aug 27;16(9):1509. doi: 10.3390/bs16091509 (PMC13603259; doi:10.3390/bs16091509)
Supplement: Supplementary file 1 [file behavsci-16-01509-s001.zip › behavsci-4382266-supplementary.pdf]

Supplementary Material S1. Retrospective Bullying Questionnaire (RBQ)

The Retrospective Bullying Questionnaire (RBQ) used in the present study was translated into Chinese by our research team following a standard translation and cultural adaptation procedure. The Chinese version was subsequently administered to 3,859 Chinese college students to evaluate its psychometric properties. The questionnaire consists of 21 items covering four dimensions. All items were rated on a 5-point Likert scale ranging from 1 (Never) to 5 (Always). Higher scores indicate more severe bullying victimization experiences. The complete questionnaire is provided below.

Table S1. RBQ Items

Item	
Were you physically bullied during elementary school?	——Being hit, pushed, kicked, or shoved<br>——Having money or possessions stolen, robbed, extorted, or deliberately damaged
Were you verbally bullied during elementary school?	——Being called names, teased, or made fun of<br>——Being threatened or intimidated
Were you indirectly bullied during elementary school?	——Having rumors spread about you or being talked about behind your back<br>——Being excluded or ostracized by others
Were you physically bullied during secondary school?	——Being hit, pushed, kicked, or shoved<br>——Having money or possessions stolen, robbed, extorted, or deliberately damaged
Were you verbally bullied during secondary school?	——Being called names, teased, or made fun of<br>——Being threatened or intimidated
Were you indirectly bullied during secondary school?	——Having rumors spread about you or being talked about behind your back<br>——Being excluded or ostracized by others
Do you have clear, distressing memories of the bullying incident(s) that repeatedly come to mind?	
Do you have dreams or nightmares about the bullying incident(s)?	
Have you ever felt as if you were reliving the bullying incident(s)?	
Have you ever suddenly vividly recalled or experienced "flashbacks" of the bullying incident(s)?	
Do you feel emotionally distressed when you think about the bullying incident(s)?	
Did you have pleasant experiences during elementary school?	
Did you have pleasant interactions with your family during elementary school?	
Did you have pleasant experiences during secondary school?	
Did you have pleasant interactions with your family during secondary school?	

Table S2. Reliability Analysis of RBQ Subscales (N = 3,859)

	rumination	primary school victimization	secondary school victimization	positive experiences
Cronbach's	0.95	0.90	0.92	0.83
McDonald's	0.99	0.91	0.93	0.83
Split-half	0.95	0.89	0.92	0.74
Test-retest	0.94	0.88	0.93	0.88
